# Supplementary figures and images for: Deficiency in the autophagy modulator Dram1 exacerbates pyroptotic cell death of Mycobacteria-infected macrophages
Source: Cell Death Dis. 2020 Apr 24;11(4):277. doi: 10.1038/s41419-020-2477-1 (PMC7181687; doi:10.1038/s41419-020-2477-1)

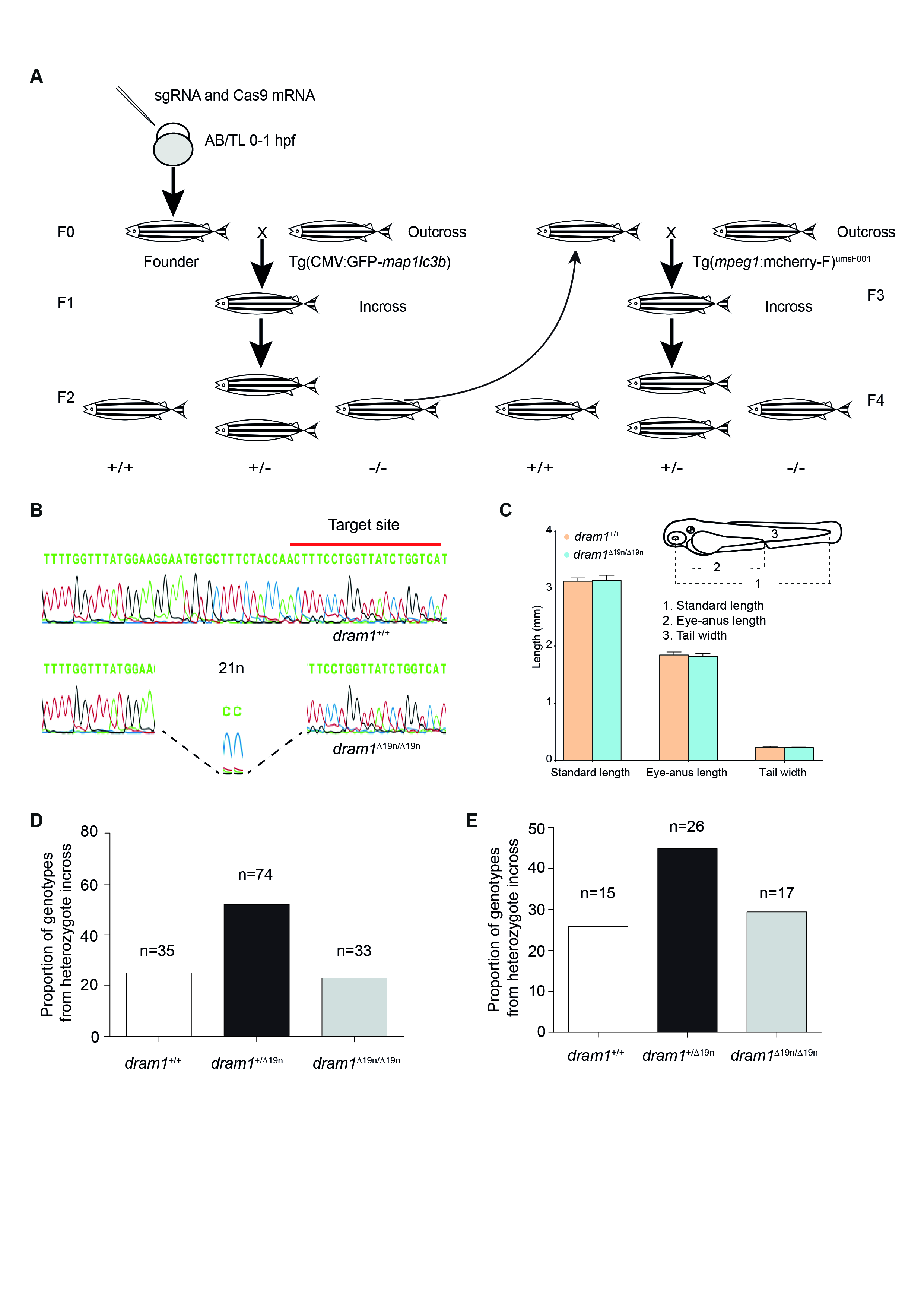

Supplement: Supplementary file 1 — Supplemental Figure 1 [file 41419_2020_2477_MOESM1_ESM.tif]

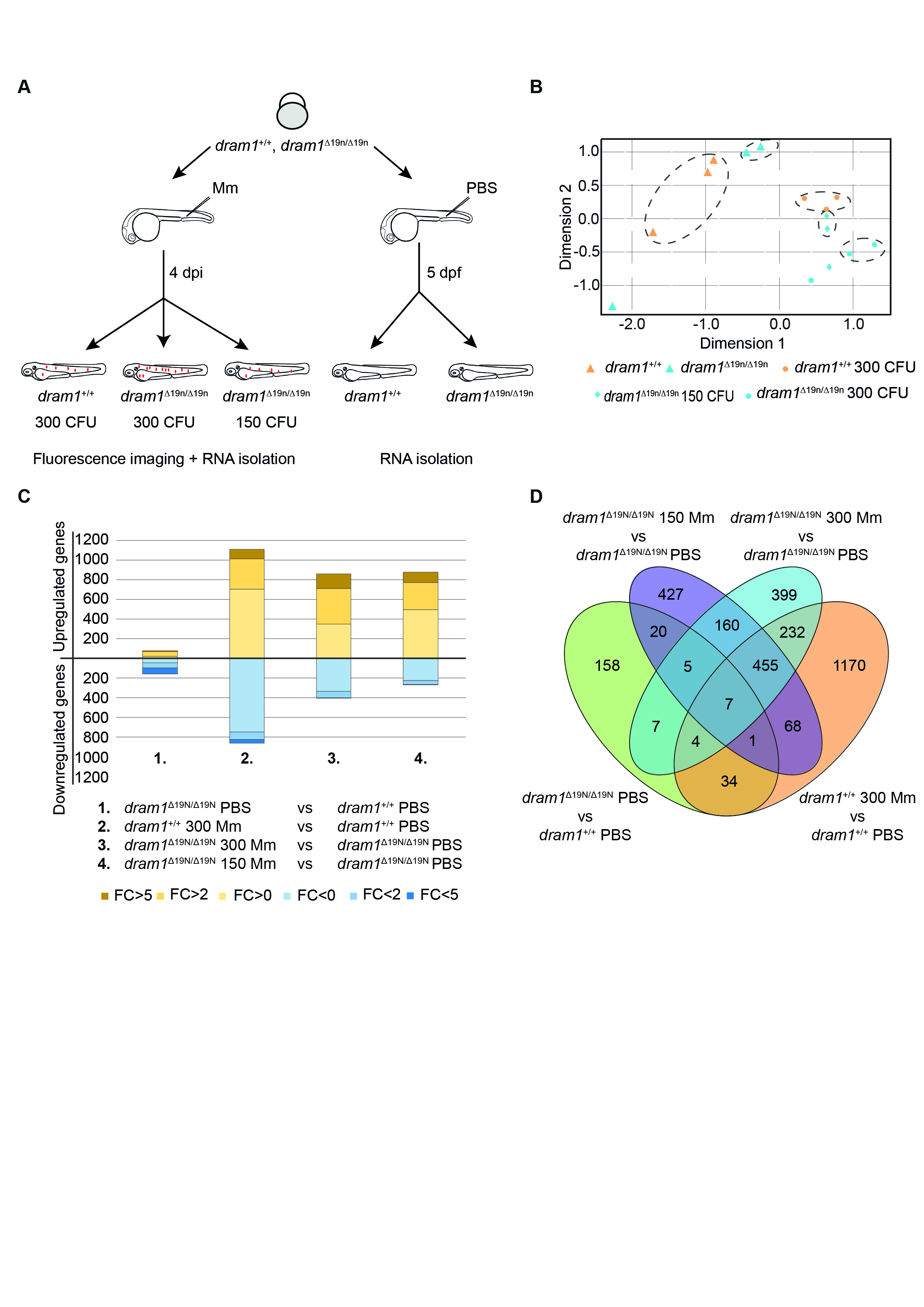

Supplement: Supplementary file 2 — Supplemental Figure 2 [file 41419_2020_2477_MOESM2_ESM.tif]

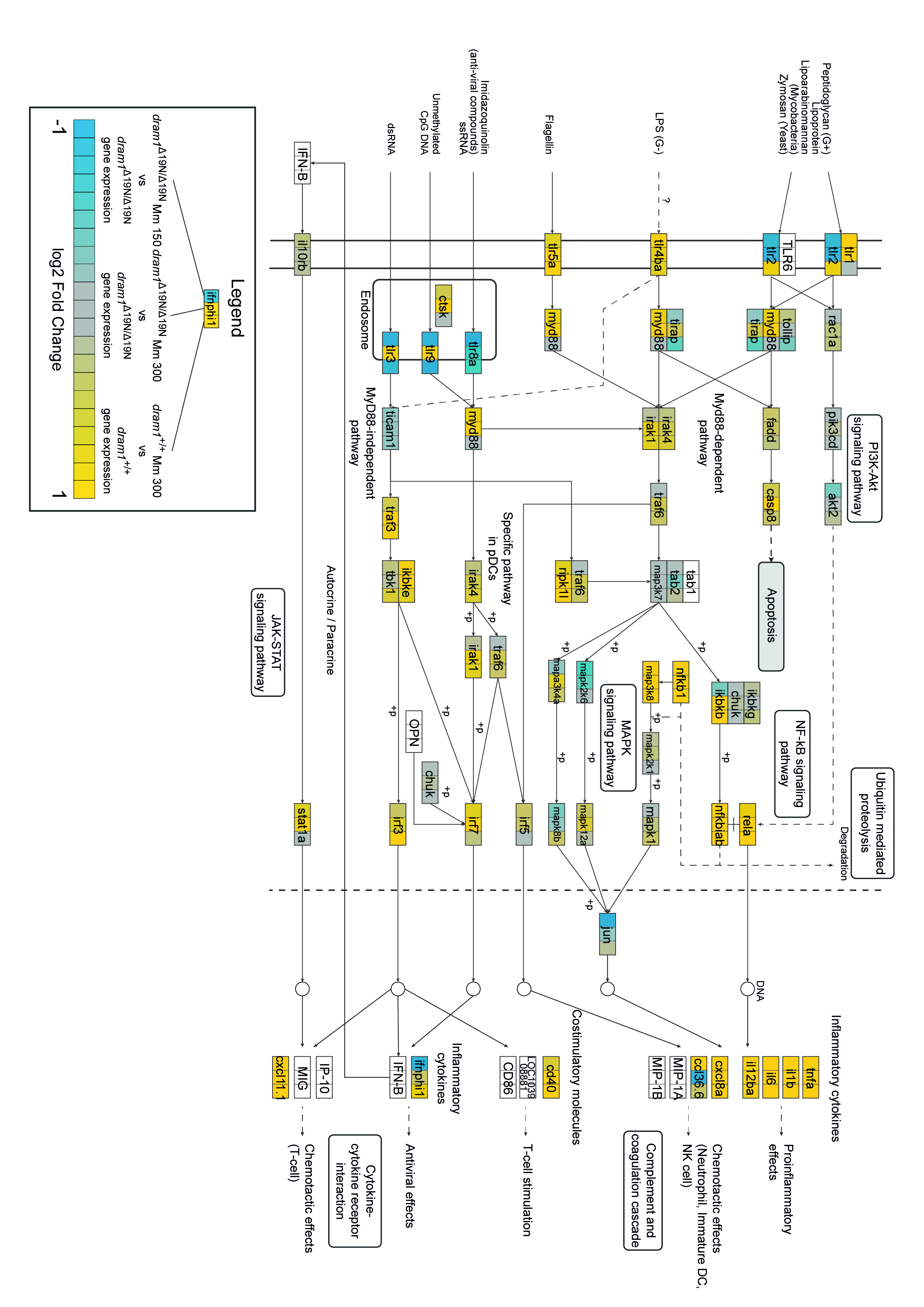

Supplement: Supplementary file 3 — Supplemental Figure 3 [file 41419_2020_2477_MOESM3_ESM.tif]
