# Supplementary material for: Deficiency in the autophagy modulator Dram1 exacerbates pyroptotic cell death of Mycobacteria-infected macrophages
Source: Cell Death Dis. 2020 Apr 24;11(4):277. doi: 10.1038/s41419-020-2477-1 (PMC7181687; doi:10.1038/s41419-020-2477-1)
Supplement: Supplementary file 5 — Supplemental Table 2 [file 41419_2020_2477_MOESM5_ESM.docx]

**Supplementary Table 2. Zebrafish lines used in this study**

| Name | Description | Reference |
| --- | --- | --- |
| AB/TL | Wild type strain | 6 |
| *Tg*(*CMV:EGFP-map1lc3b*) | GFP reporter transgenic zebrafish for Lc3 | 26 |
| *Tg*(*mpeg1:mCherryF*)*^umsF001^* | Macrophage marker with membrane-localizing *mCherryF* | 27 |
| *dram1*^+/+^/GFP-Lc3 | Siblings of *dram1 ^ibl53^* carrying a transgenic GFP-Lc3 reporter | In this study |
| *dram1*^∆19n/∆19n^/GFP-Lc3 | *dram1^ibl53^*mutant line (∆19n indel) carrying a transgenic GFP-Lc3 reporter | In this study |
| *dram1*^+/+^/*mpeg1:mCherryF* | Siblings of *dram1 ^ibl53^* mutant line carrying a transgenic *mpeg1:mCherryF* reporter | In this study |
| *dram1*^∆19n/∆19n^/*mpeg1:mCherryF* | *dram1^ibl53^*mutant line (∆19n indel) carrying a transgenic *mpeg1:mCherryF* reporter | In this study |
