# Supplementary material for: Deficiency in the autophagy modulator Dram1 exacerbates pyroptotic cell death of Mycobacteria-infected macrophages
Source: Cell Death Dis. 2020 Apr 24;11(4):277. doi: 10.1038/s41419-020-2477-1 (PMC7181687; doi:10.1038/s41419-020-2477-1)
Supplement: Supplementary file 6 — Supplemental Table 3 [file 41419_2020_2477_MOESM6_ESM.docx]

**Supplementary Table 3. Primers for complementation and amplification of sgRNA**

| Name | Forward (5’-3’) | Reverse (5’-3’) |
| --- | --- | --- |
| Dram1 sgRNA template | GCG**TAATACGACTCACTATAG**GACCAGATAACCAGGAAAGTTGGTTTTAGAGCTAGAAATAGCAAGTTAAAATAAGGCTAGTC | GATCCGCACCGACTCGGTGCCACTTTTTCAAGTTGATAACGGACTAGCCTTATTTTAACTTGCTATTTCTAGCTCTAAAAC |
| sgRNA amplify | GCGTAATACGACTCACTATAG | GATCCGCACCGACTCGGT |

* T7 promoter: 5’-**TAATACGACTCACTATAG**-3’; The underlined sequence indicates the target sites for gRNAs designed
