# Supplementary material for: Deficiency in the autophagy modulator Dram1 exacerbates pyroptotic cell death of Mycobacteria-infected macrophages
Source: Cell Death Dis. 2020 Apr 24;11(4):277. doi: 10.1038/s41419-020-2477-1 (PMC7181687; doi:10.1038/s41419-020-2477-1)
Supplement: Supplementary file 7 — Supplemental Figure Legends [file 41419_2020_2477_MOESM7_ESM.docx]

## **Supplementary Figure Legends**

**Figure S1: Generation and characterization of the *dram1* mutant line.**

A. Schematic diagram showing the workflow used for the generation of *dram1* mutant lines. Target-specific sgRNA and Cas9 mRNA were co-injected into one-cell stage embryos (AB/TL, wild type line). Founders were outcrossed to *Tg*(*CMV:EGFP-map1lc3b*) or wild type fish to obtain F1. After 3-4 months, the F1 was incrossed to obtain homozygous mutant and wild type F2 siblings. *dram1*^∆19n/∆19n^ were outcrossed with the macrophage marker *Tg*(*mpeg1:mCherryF*)*^umsF001^* and after 3-4 months subsequently incrossed to obtain *dram1^+/+^*, *dram1*^∆19/+^, and *dram1*^∆19n/∆19n^ carrying *Tg*(*mpeg1:mCherryF*)*^umsF001^*.

B. Sanger sequencing of *dram1*^∆19n/∆19n^  and *dram1^+/+^* from F2 offspring. The red line indicates CRISPR/Cas9 target sites. The genomic DNA was isolated from fin tissue (>3 months old fish). The *dram1*^∆19n/∆19n^ mutant allele has 21 nucleotides deleted and 2 nucleotides inserted.

C. Measurements of larval body lengths. *dram1*^+/+^ and *dram1*^∆19n/∆19n^ larvae (≥10 larvae/group) were imaged by stereo microscopy at 3dpf and body lengths were measured as indicated by the red dotted lines.

D. Segregation from *dram1*^+/∆19n^ F1 heterozygous incross. Genotypes of adult fish (>3 months old) were combined from at least three independent breedings and confirmed by PCR and Sanger sequencing. Data were analyzed by Chi-square test. ns, non-significant,*p<0.05,**p<0.01,***p<0.001.

E. Segregation from *dram1*^∆19n+^/*mpeg1:mCherryF* F1 heterozygous incross. Genotypes of adult fish (>3 months old) combined from at least three independent breedings were confirmed by PCR and sequencing. Data were analyzed by Chi-square test. ns, non-significant,*p<0.05,**p<0.01,***p<0.001.

**Figure S2: Transcriptome analysis of uninfected and infected *dram1* mutants**

A. Experimental design to obtain samples for RNA deep sequencing. *Mycobacterium marinum* strain M (Mm) fluorescently labeled with mCherry was microinjected into the blood island of embryos at 28 hpf at an injection dosage of 300 CFU or 150 CFU. Control groups were injected with PBS.

B. Principal component analysis of the gene expression data obtained by RNA sequencing. The RNA sequencing samples clustered according to their condition, as pictured by the dashed ellipses grouping the samples. The data sets of one family of *dram1*^∆19n/∆19n^ (Mm infected and uninfected) diverged from the rest of the libraries (data points outside the dashed ellipses) and were discarded from the further analysis.

C. Overall profile of differential gene expression between the different conditions. The number of genes upregulated is depicted in yellow bars while the number of downregulated genes are depicted in blue, with indication of the fold-change by color intensity.

D. Venn diagram of the differentially expressed genes common and different between the *dram1*^∆19n/∆19n^ and *dram1*^+/+^, *dram1*^∆19n/∆19n^150 CFU and *dram1*^∆19n/∆19n^ PBS, *dram1*^∆19n/∆19n^ 300 CFU and *dram1*^∆19n/∆19n^, and *dram1*^+/+^ 300 CFU and *dram1*^+/+^ PBS comparisons.

**Figure S3: Effect of *dram1* mutation on TLR signaling**

KEGG pathway of TLR signaling showing differential gene expression in infected *dram1*^∆19n/∆19n^ and *dram1*^+/+^. The three data sets used for comparison are shown in the legend of the figure. The expression fold change of the genes is depicted by color (yellow: upregulated, blue: downregulated).

**Supplementary tables**

**Supplementary Table 1. Enrichment of gene sets altered in *dram1*^∆19n/∆19n^ larvae under basal conditions.**

A. Gene Ontology categories significantly over and underrepresented in the significant genes differentially regulated between *dram1*^∆19n/∆19n^ PBS-injected mutants compared to *dram1*^+/+^ larvae.

B. Gene sets from the MSigDB C2 database significantly positively correlated to the *dram1*^∆19n/∆19n^ mutants transcriptome compared to *dram1*^+/+^ larvae.

C. Gene sets from the MSigDB C2 database significantly negatively correlated to the *dram1*^∆19n/∆19n^ mutants transcriptome compared to *dram1*^+/+^ larvae.

For data set see: https://doi.org/10.5281/zenodo.2615900
